# Supplementary material for: Magnetosomes and Magnetosome Mimics: Preparation, Cancer Cell Uptake and Functionalization for Future Cancer Therapies
Source: Pharmaceutics. 2021 Mar 10;13(3):367. doi: 10.3390/pharmaceutics13030367 (PMC7998144; doi:10.3390/pharmaceutics13030367)
Supplement: Supplementary file 1 [file pharmaceutics-13-00367-s001.pdf]

pharmaceutics

MDPI

Supplementary Materials: Magnetosomes and Magnetosomes Mimics: Preparation, Cancer Cell Uptake and Functionalization for Future Cancer Therapies

Zainab Taher, Christopher Legge, Natalie Winder, Pawel Lysyganicz, Andrea Rawlings, Helen Bryant, Munitta Muthana and Sarah Staniland

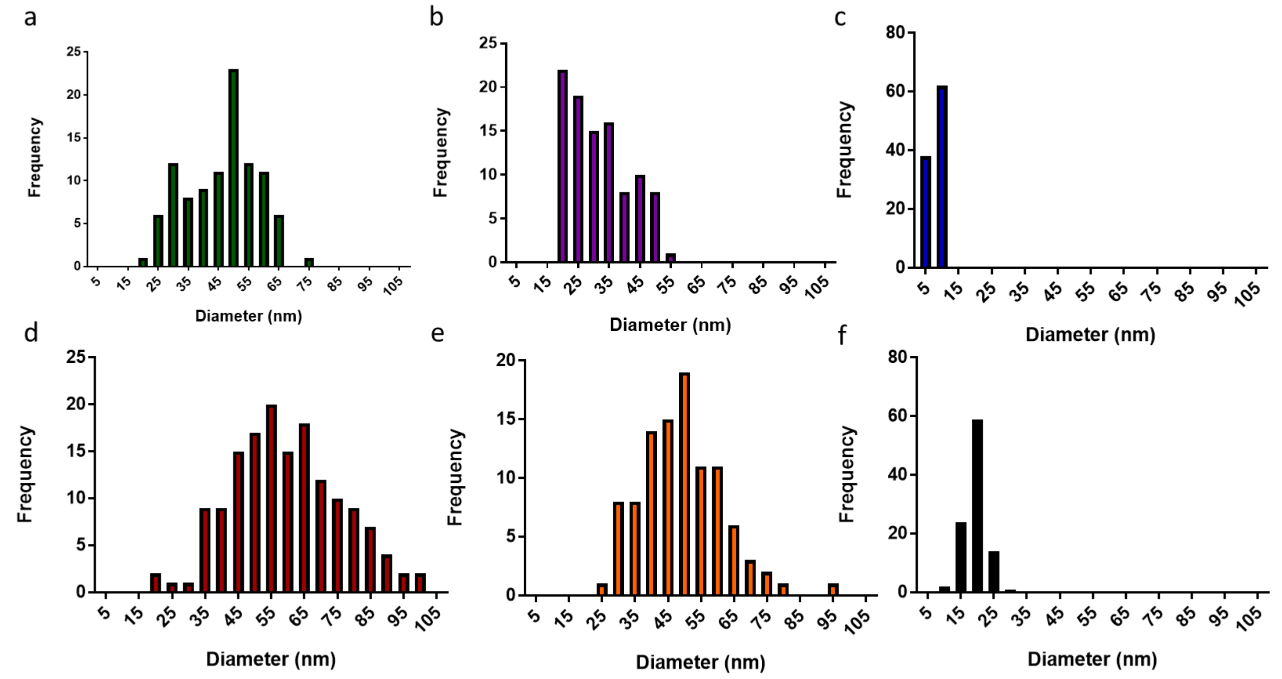

Figure S1. Histograms of synthesised magnetosomes and MNPs showing the size distribution of the particles measured from images taken via TEM, image analysis was performed on Image J software. (a) magnetosomes, (b) cMNP, (c) rMNP, (d) OA@cMNP, (e) Si@cMNP, (f) Si@rMNP.

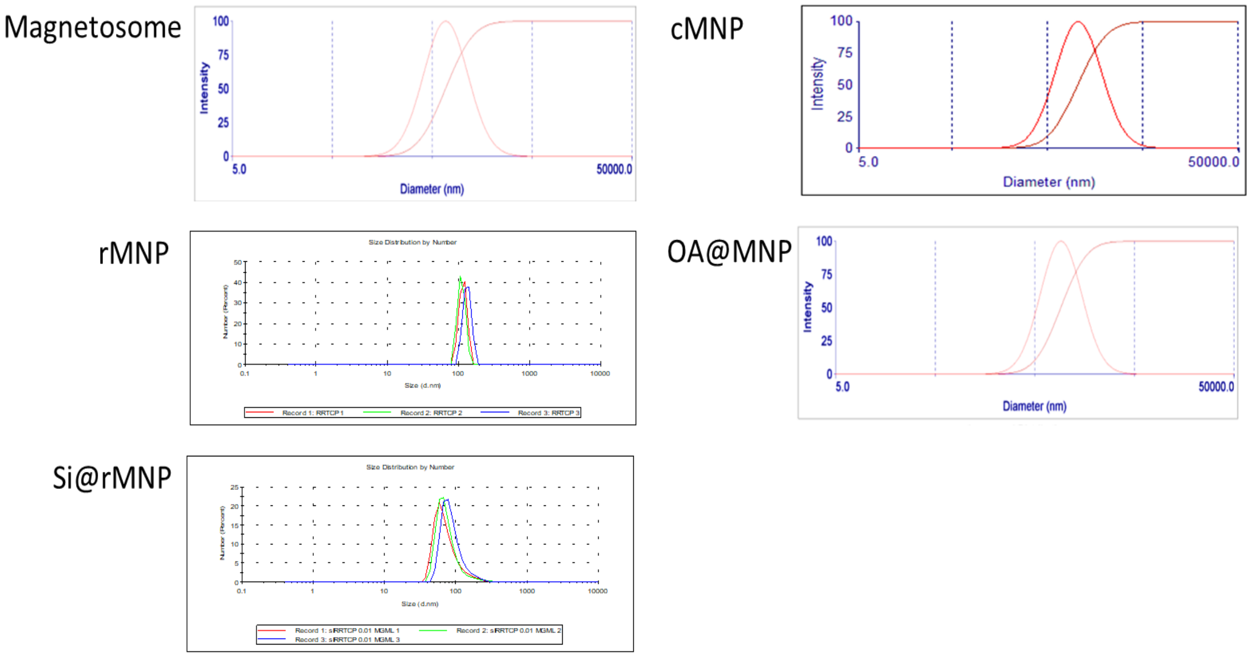

Figure S2. Hydrodynamic size measurements showing the size that the particles appear in solution performed via dynamic light scattering (DLS) of the magnetosomes and MNPs at a 0.01 mg ml concentration dispersed in milliq. .
